# Cytokine levels associated with favorable clinical outcome in the CAPSID randomized trial of convalescent plasma in patients with severe COVID-19

**DOI:** 10.3389/fimmu.2022.1008438

**Published:** 2022-10-06

**Authors:** Sixten Körper, Eva Vanessa Schrezenmeier, Hector Rincon-Arevalo, Beate Grüner, Daniel Zickler, Manfred Weiss, Thomas Wiesmann, Kai Zacharowski, Johannes Kalbhenn, Martin Bentz, Matthias M. Dollinger, Gregor Paul, Philipp M. Lepper, Lucas Ernst, Hinnerk Wulf, Sebastian Zinn, Thomas Appl, Bernd Jahrsdörfer, Markus Rojewski, Ramin Lotfi, Thomas Dörner, Bettina Jungwirth, Erhard Seifried, Daniel Fürst, Hubert Schrezenmeier

**Affiliations:** ^1^ Institute for Clinical Transfusion Medicine and Immunogenetics Ulm, German Red Cross Blood Transfusion Service Baden-Württemberg-Hessen and University Hospital Ulm and Institute of Transfusion Medicine, University of Ulm, Ulm, Germany; ^2^ Department of Nephrology and Medical Intensive Care, Charité - Universitätsmedizin Berlin, corporate member of Free University Berlin, Humboldt-Universität zu Berlin, and Berlin Institute of Health, Berlin, Germany; ^3^ Berlin Institute of Health Charité Universitätsmedizin Berlin, Berlin Institute of Health (BIH) Academy, Berlin, Germany; ^4^ Grupo de Inmunología Celular e Inmunogenética, Facultad de Medicina, Instituto de Investigaciones Médicas, Universidad de Antioquia UdeA, Medellín, Colombia; ^5^ Division of Infectious Diseases, University Hospital and Medical Center Ulm, Ulm, Germany; ^6^ Department of Anaesthesiology and Intensive Care Medicine, University Hospital Ulm, Ulm University, Ulm, Germany; ^7^ Department of Anaesthesiology and Intensive Care Medicine, Phillips-University Marburg, Marburg, Germany; ^8^ Clinic of Anaesthesiology, Intensive Care Medicine and Pain Therapy, University Hospital Frankfurt, Frankfurt, Germany; ^9^ Clinic of Anesthesiology and Intensive Care Medicine University Medical Center of Freiburg, Freiburg, Germany; ^10^ Department of Internal Medicine III, Hospital of Karlsruhe, Karlsruhe, Germany; ^11^ Medical Clinic I, Klinikum Landshut, Landshut, Germany; ^12^ Department of Gastroenterology, Hepatology, Pneumology and Infectious Diseases, Klinikum Stuttgart, Stuttgart, Germany; ^13^ Department of Internal Medicine V – Pneumology, Allergology, Intensive Care Medicine, Saarland University Hospital, Homburg, Germany; ^14^ Department of Rheumatology and Clinical Immunology, Charité Universitätsmedizin Berlin, Berlin, Germany; ^15^ Deutsches Rheumaforschungszentrum (DRFZ), Berlin, Germany; ^16^ Institute of Transfusion Medicine and Immunohematology, German Red Cross Blood Transfusion Service Baden-Württemberg – Hessen, Frankfurt, Germany

**Keywords:** COVID-19, randomized trial, convalescent plasma, predictive factors, chemokines, interleukins

## Abstract

**Objectives:**

To determine the profile of cytokines in patients with severe COVID-19 who were enrolled in a trial of COVID-19 convalescent plasma (CCP).

**Methods:**

Patients were randomized to receive standard treatment and 3 CCP units or standard treatment alone (CAPSID trial, ClinicalTrials.gov NCT04433910). The primary outcome was a dichotomous composite outcome (survival and no longer severe COVID-19 on day 21). Time to clinical improvement was a key secondary endpoint. The concentrations of 27 cytokines were measured (baseline, day 7). We analyzed the change and the correlation between serum cytokine levels over time in different subgroups and the prediction of outcome in receiver operating characteristics (ROC) analyses and in multivariate models.

**Results:**

The majority of cytokines showed significant changes from baseline to day 7. Some were strongly correlated amongst each other (at baseline the cluster IL-1ß, IL-2, IL-6, IL-8, G-CSF, MIP-1α, the cluster PDGF-BB, RANTES or the cluster IL-4, IL-17, Eotaxin, bFGF, TNF-α). The correlation matrix substantially changed from baseline to day 7. The heatmaps of the absolute values of the correlation matrix indicated an association of CCP treatment and clinical outcome with the cytokine pattern. Low levels of IP-10, IFN-γ, MCP-1 and IL-1ß on day 0 were predictive of treatment success in a ROC analysis. In multivariate models, low levels of IL-1ß, IFN-γ and MCP-1 on day 0 were significantly associated with both treatment success and shorter time to clinical improvement. Low levels of IP-10, IL-1RA, IL-6, MCP-1 and IFN-γ on day 7 and high levels of IL-9, PDGF and RANTES on day 7 were predictive of treatment success in ROC analyses. Low levels of IP-10, MCP-1 and high levels of RANTES, on day 7 were associated with both treatment success and shorter time to clinical improvement in multivariate models.

**Conclusion:**

This analysis demonstrates a considerable dynamic of cytokines over time, which is influenced by both treatment and clinical course of COVID-19. Levels of IL-1ß and MCP-1 at baseline and MCP-1, IP-10 and RANTES on day 7 were associated with a favorable outcome across several endpoints. These cytokines should be included in future trials for further evaluation as predictive factors.

## Introduction

Clinical course of COVID-19 can vary greatly. It has been shown that hyperinflammation is a hallmark of progression to severe COVID-19. Plasma levels of many proinflammatory cytokines are elevated in COVID-19 patients, e.g. IL-1ß, IL-2, IL-4, IL-5, IL-6, IL-7, IL-8, IL-10, IL-13, IL-15, IL-17, colony-stimulating factors (G-CSF), granulocyte-macrophage stimulating factor (GM-CSF), interferon-inducible protein 10 (IP-10), interferon-α (IFN-α), interferon-γ (IFN-γ), tumor necrosis factor-α (TNF-α), vascular endothelial growth factor (VEGF), and various chemokines MCP-1 (CCL-2), MIP-1α (CCL3) or RANTES (CCL5) (1–9). While many publications report on the hyperinflammatory response in real-world patient series, limited information on cytokine pattern is available from randomized clinical trials of anti-SARS-CoV-2 drugs (2).

Here we report a comprehensive analysis of the cytokine pattern which was analyzed in a companion research project in a randomized clinical trial of COVID-19 convalescent plasma (CCP) for severe COVID-19 (CAPSID trial, NCT04433910, EudraCT 2020-001310-38) (10). In this trial patients were randomized 1:1 to either receive standard treatment and 3 units of CCP or standard treatment alone. One CCP unit each was transfused to patients in the CCP group on day +1, +3 and +5. Primary outcome was a dichotomous composite outcome of survival and no longer fulfilling criteria for severe COVID-19 on day 21. The key secondary outcome was the time to clinical improvement which was defined as improvement by at least two points on the ordinal severity scale (11). Clinical results of this trial have been previously published (10). The primary outcome occurred in 43.4% of patients in the CCP and 32.7% in the control group (p=0.32). The median time to clinical improvement was 26 days in the CCP group and 66 days in the control group (p=0.27). In the subgroup that received a higher cumulative amount of neutralizing antibodies the primary outcome occurred in 56.0% (versus 32.1%), with significantly shorter interval to clinical improvement (20 versus 66 days)(p<0.05) and better overall survival (day-60 probability of survival 91.6% versus 68.1%; p=0.02) compared to the control group (10).

The serum concentrations of a panel of 27 interleukins, chemokines and growth factors (hereafter referred to as cytokines) were measured at baseline and on day 7: IL-1ß, IL-1RA, IL-2, IL-4, IL-5, IL-6, IL-7, IL-8, IL-9, IL-10, IL-12 (p70), IL-13, IL-15, IL-17, IFN-γ, IP-1ß, TNF-α, Eotaxin, VEGF, PDGF-BB, GM-CSF, G-CSF, bFGF, RANTES, MCP-1, MIP-1α and MIP-1β. We analyzed the change of cytokines over time, the correlation matrix among these 27 cytokines and their association with the primary endpoint (i.e. survival and no longer COVID-19 on day +21) and the key secondary endpoint (time to clinical improvement) of the CAPSID trial.

## Methods

Patients: A total of 106 patients were enrolled to the clinical trial CAPSID and randomized to receive standard treatment alone (control group) or 3 CCP units and standard treatment (CCP group) (10). Patients in the CCP group received one CCP unit each on days +1, +3 and +5 with a median total volume 846 ml (IQR 824-855 ml). 53 patients were included in this study of cytokine levels as inflammation markers, 22 in the control group and 31 in the CCP group. Clinical characteristics of the patients are summarized in **Table 1**. Those patients were included in this analysis who had an available serum sample at both baseline and day 7.

**Table 1:** Baseline demographics and clinical characteristics.

	CCP group (n=31)	Control Group (n=22)	p-value
**Demographic and clinical characteristics**			
**Age (years), median (IQR)**	58 (52-63)	58.5 (51.3-67.5)	0.82
**Gender, no (%)**			0.29
** Female**	4 (12.9)	6 (27.3)	
** Male**	27 (87.1)	16 (72.7)	
**ABO Blood group, n (%)**			0.80
** 0**	12 (38.7)	6 (27.3)	
** A**	14 (45.2)	12 (54.6)	
** AB**	2 (6.5)	1 (4.6)	
** B**	3 (9.7)	3 (13.6)	
Inflammation marker* (n,%)			0.48
** low**	17 (54.8)	11 (50.0)	
** intermediate**	0 (0.0)	1 (4.6)	
** high**	14 (45.2)	10 (45.6)	
**WHO Point Scale, n (%)**			0.89
** 3**	3 (9.7)	2 (9.1)	
** 4**	5 (16.1)	5 (22.7)	
** 5**	21 (67.7)	14 (63.6)	
** 6**	1 (3.2)	0 (0.0)	
** 7**	1 (3.2)	1 (4.6)	
SARS-CoV-2 antibody status*, n (%)			0.84
**Neutralizing antibodies**			
** positive**	22 (71.0)	16 (72.7)	
** negative**	6 (19.4)	5 (22.7)	
** missing**	3 (9.6)	1 (4.5)	

*for definition see Material and Methods.

### Outcome measures

Outcome measures for the primary and secondary outcome have been previously reported (10). The primary outcome of the CAPSID trial (“treatment success”) was assessed on day 21 after randomization and is a dichotomous composite outcome of survival and no longer requiring ventilation support or ICU treatment and no tachypnea (i.e., respiratory rate <30 breaths/minute) on day 21. The key secondary outcome time to clinical improvement was defined as an increase by at least two points on the ordinal WHO severity scale (11). Patients without documented improvement were censored at last follow up. The scale was defined as follows: 0, no clinical or virological evidence of infection; 1, ambulatory without limitation of activities; 2, ambulatory with limitation of activities; 3, hospitalized without oxygen therapy; 4, hospitalized with supplemental oxygen by mask or nasal prongs; 5, hospitalized, non-invasive ventilation or high-flow oxygen; 6, hospitalized, intubation and mechanical ventilation; 7, hospitalized, ventilation and additional organ support (vasopressors, renal replacement therapy or ECMO); 8, death. Further secondary outcomes were mortality; duration of ventilation support; time to discharge from ICU; time to hospital discharge; time until negative SARS-CoV-2 PCR from a nasopharyngeal swab. Survival time was time from randomization to death in days. Patients who died during the observation period without reaching the secondary outcome were censored as if they had reached the end of observation to account for the competing risk setting. The primary and secondary outcomes were also analyzed in a subgroup analysis by transfused neutralizing units. Since the total amount of neutralizing antibodies depends on both the volume and the antibody titer of CCP we used “neutralizing units” to take into account both variables. One neutralizing unit was arbitrarily defined as one ml of CCP with a titer of 1:20 which achieves 50% inhibition in the plaque reduction neutralization test (PRNT50). The neutralizing units of a CCP transfusion unit were then calculated by dividing the titer by 20 and multiplying by volume (ml) (10). CCP group was divided by the cumulative amount of neutralizing units per patient (all 3 CCP transfusions) in a low neutralizing unit group (≤ median) and a high neutralizing unit group (> median).

One of the trial endpoints was to analyze the predictive value of inflammation on clinical improvement, mortality, length of stay in ICU and length of hospital stay which is presented in this manuscript.

### Plaque reduction neutralization test for SARS-CoV-2

Plaque reduction neutralization tests for SARS-CoV-2 were performed as previously described (12–14). Briefly, VeroE6 cells (3.25x10^5^ cell/ml) were seeded in 24-well plates and incubated overnight. Prior to PRNT, patient sera were heat-inactivated at 56°C for 30 minutes. For each dilution step (duplicate), patient sera were diluted in 220 μl OptiPro and mixed 1:1 with 220 μl virus solution containing 100 plaque forming units. The 440 μl serum-virus solution was gently vortexed and incubated at 37°C for 1 hour. Each 24-well was incubated with 200 μl serum-virus solution. After 1 hour at 37°C supernatants were discarded, and cells were supplemented with 1.2% Avicel solution in DMEM. After 3 days at 37°C, supernatants were removed and the 24-well plates were fixed and inactivated using a 6% formaldehyde/PBS solution and stained with crystal violet as described (13, 14). Serum dilutions with a plaque reduction of 50% (PRNT50) and 90% (PRNT90) are referred to as titers. Unless stated otherwise, cut off titers were set at < 1:20. Positive neutralizing antibody status means a PRNT50 titer ≥ 1:20, a negative status means < 1:20.

### Study approval

The clinical trial CAPSID was approved by the Federal Authority Paul-Ehrlich-Institute and by the Ethical Committee of the University of Ulm and the ethical committees of the participating hospitals. The CAPSID trial is registered: EudraCT number 2020-001310-38 and NCT04433910. Written informed consent was obtained from all study participants or their legal representatives.

### Cytokine measurements

Serum cytokine levels were determined using the human Bio-Plex Pro Human Cytokine 27-plex Assay (Bio-Rad, Hercules, USA) containing the following cytokines: IL-1ß, IL-1RA, IL-2, IL-4, IL-5, IL-6, IL-7, IL-8, IL-9, IL-10, IL-12 (p70), IL-13, IL-15, IL-17, IFN-γ, IP-1ß, TNF-α, Eotaxin, VEGF, PDGF-BB, GM-CSF, G-CSF, bFGF, RANTES, MCP-1, MIP-1α and MIP-1β. The panel was chosen to include the following: type 1 cytokines (IL-2, IL-12, IFN-γ); type 2 cytokines (IL-4, IL-5, IL-6, IL-9, IL-10, IL-13); Th17 cytokines (IL-17); chemokines indicating activation of the innate immune system or endothelial cells (MCP-1, MIP-1α, MIP-1ß, RANTES, eotaxin, IP-10), factors indicating inflammosome activation (IL-1ß) and factors stimulating growth and development of hematopoietic cells, immune cells, endothelial cells and mesenchymal cells (G-CSF, GM-CSF, PDGF, IL-7, VEGF, bFGF). This selection covers a broad range of adaptive immunity cytokines, pro-inflammatory cytokines, and anti-inflammatory cytokines and growth stimulating factors to enable an unbiased, comprehensive analysis of a broad range of extracellular signaling interactions. This panel partially overlaps with cytokines chosen in other COVID-19 marker studies to allow a comparison of this analysis in the setting of a controlled clinical trial with other published cohort studies (5–7, 15–19).

For the measurement serum samples were processed in accordance with the protocol provided by the manufacturer. In short, standards and samples were diluted (1:4) in sample diluent and transferred to the plate containing magnetic beads. The plate was washed and the detection antibody was added. The plate was washed again and streptavidin-PE solution was added. After washing again, the samples were resuspended in 125 µL of assay buffer and analyzed within 15 min using a Bio-Plex 200 system reader system (Bio-Rad, Hercules, USA).

### Statistics

Since the data set was complete for the 53 patients with day 0 and day 7 information including all 27 cytokines no imputation for missing variables was performed.

Groups were compared using either Kruskal-Wallis test with Dunn´s *post hoc* test or nonparametric two-tailed Mann-Whitney test. The horizontal lines in the scatter plots of cytokine levels indicate the median ± interquartile range (IQR). Differences were considered significant at p<0.05. The asterisk in the graphs indicate the following significance levels: *p<0.05, ** p<0.01, *** p<0.001, **** p<0.0001.

The scatter plots and comparisons between groups were done with GraphPad Prism 9 for Windows (Version 9.0.3).

The association between levels of different cytokines are presented as heat maps showing the absolute values of the correlation matrix. Rows and columns in the heat maps are sorted in the order suggested by a hierarchical clustering of the correlation matrix. The list of variables contained in these clusters is reported in **Tables 2A**, **2B**. Those cytokines that could not be classified are listed in the “none” clusters. Cophenetic correlations of all hierarchical cluster analyses presented were 0.75 or above. The unweighted pair group method was used for clustering.

**Table 2A:** Cluster report for the absolute values of the correlation matrix of all patients on day 0 and day 7.

Cluster*	day 0: all patients		Day 7: all patients	
1	IL-1ß, IL-2, IL-6, IL-8, G-CSF, MIP-1α		IL-1ß, MIP-1α	
2	PDGF-BB, RANTES		IL-1RA, IL-5, IFN-γ, IP-10, MCP-1	
3	IL-4, IL-17, Eotaxin, bFGF, TNF-α		IL-2, IL-4, IL-17, Eotaxin, bFGF, MIP-1ß, TNF-α	
4	IL-5, IFN-γ, MCP-1		IL-6, IL-10, G-CSF	
5	IL-9, MIP-1ß		IL-15, VEGF	
6	IL-15, VEGF		IL-9, RANTES	
7			IL-12, GM-CSF	
None	IL-1RA, IL-7, IL-10, IL-12, IL-13, GM-CSF, IP-10		IL7, IL-8, IL-13, PDGF-BB	

*Clusters were formed by the unweighted group method using NCSS 2021, version 21.0.3

**Table 2B:** Cluster report for the absolute values of the correlation matrix of all patients on day 7 stratified by randomization group (control group versus CCP group) or by primary endpoint (failure versus success).

Cluster*	Day 7: control group	Day 7: CCP group	day 7: failure	Day 7: success
1	IL-1ß, MIP-1α	Il-1ß, IL-8	IL-15, VEGF	IL-1ß, G-CSF, IFN-γ, MCP, MIP-1α
2	IL-1RA, IL-5, IL-6, IFN-γ, IP-10, MCP-1	IL-1RA, IL-5, IL-6, IFN-γ, IP-10, MCP-1	IL-1RA, IL-5, IFN-γ, IP-10, MCP-1	IL-12, GM-CSF
3	IL-2, IL-4, IL-17, Eotaxin, bFGF, MIP-1ß, TNF-α	IL-2, bFGF, TNF-α	IL-2, IL-4, IL-17, Eotaxin, bFGF, MIP-1ß, TNF-α	IL-2, IL-6, IL-15, IL-17, bFGF, MIP-1ß, TNF-α
4	IL-15, VEGF	IL-4, Eotaxin	IL-6, MIP-1α	IL-4, Eotaxin
5	IL-10, G-CSF	G-CSF, MIP-1α	IL-9, RANTES	IL-5, IL-13
6		IL-9, RANTES	IL-10, G-CSF	IL-9, RANTES
7		IL-12, IL-15, GM-CSF		
		IL-17, MIP-1ß		
None	IL-7, IL-8, IL-9, IL-12, IL-13, GM-CSF, PDGF-BB, RANTES	IL-7, IL-10, IL-13, PDGF-BB, VEGF	IL-1ß, IL-7, IL-8, IL-12, IL-13, GM-CSF, PDGF-BB.	IL-1RA, IL-7, IL-8, IL-10, IP-10, PDGF-BB, VEGF.

*Clusters were formed by the unweighted group method using NCSS 2021, version 21.0.3.

The prediction of clinical outcome by cytokine levels was analyzed by receiver operating characteristics (ROC) analyses and results are presented as area under the curve (AUC) of the ROC curve and the p-value for an AUC > 0.5.

Principle component analysis (PCA), the heat maps of the absolute values of the correlation matrix and ROC analyses were performed using NCSS 2021, version 21.0.3.

The impact of treatment allocation, demographic factors, severity of COVID-19 at baseline and cytokine levels on outcome (primary outcome and key secondary outcome) was done as multivariate analysis. For primary endpoint (treatment success) logistic regression and for the secondary endpoint (time to clinical improvement) time-to event analysis using Cox-proportional hazards modeling have been used. Clinical covariates were incorporated in a stepwise backward exclusion procedure, retaining variables with a p-value <0.1 and a p-value <0.05 considered as significant. The statistical software “R” Version 4.1.2 as well as the packages “survival” version 3.2-13 and “forestmodel” version 0.6.2 have been used for multivariate analysis.

## Results

### Patients characteristics

The characteristics of the 53 patients with available samples on both baseline and day 7 are summarized in **Table 1**. The minority of patients were female (18.9%) and the majority were male (81.1%). The median age was 58 years (IQR 52-63.5). Overall, the CCP group and the control group were similar in terms of demographic characteristics and disease severity as assessed by the distribution on the ordinal severity scale, the type of ventilation support, inflammation markers and SARS-CoV-2 antibody status at baseline (**Table 1**).

### Cytokine levels on day 0 and day 7

**Figure 1:**
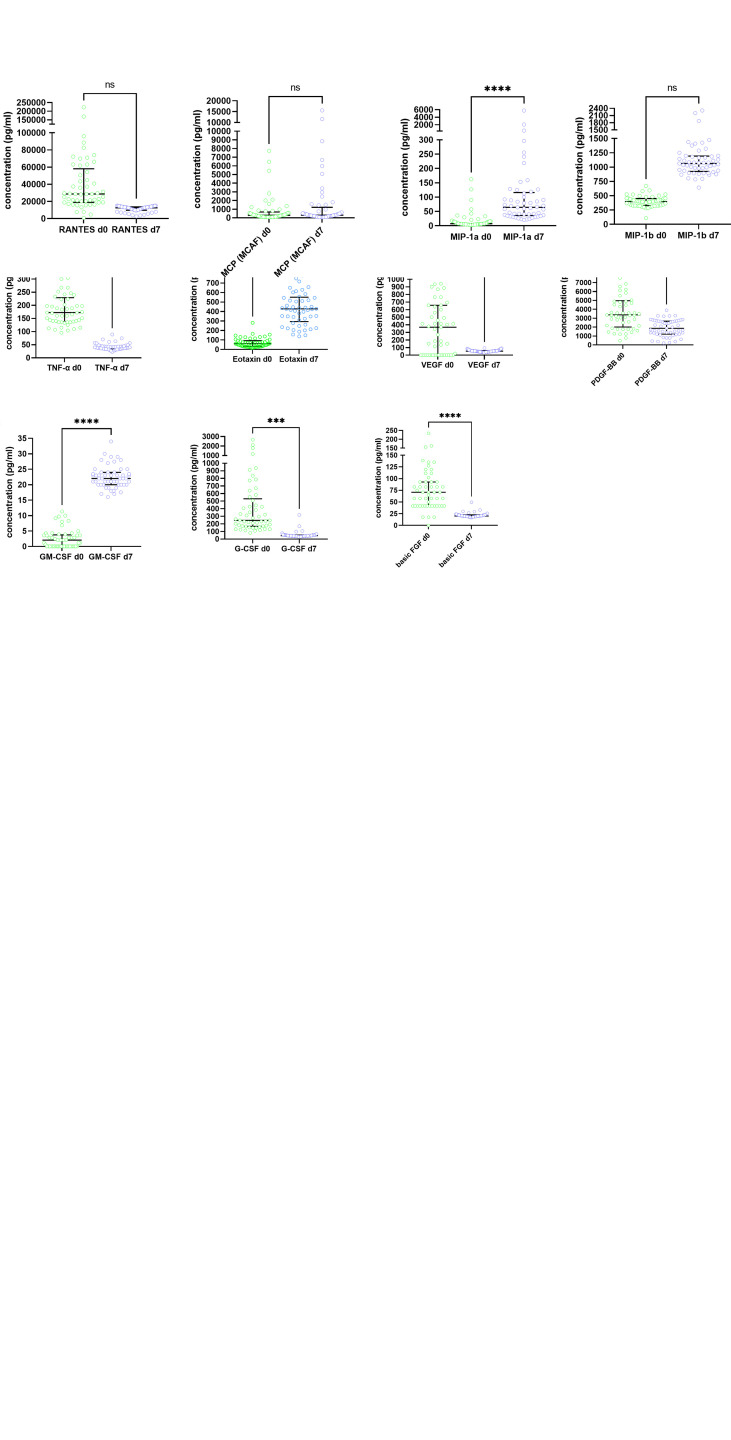
Dynamics of serum cytokine levels in the study cohort at baseline (day 0) and day 7 after randomization. Results are presented in pg/ml. Circles show individual measurements, the horizontal lines represent medians and IQR. Groups were compared by the Kruskal-Wallis-Test with Dunn´s *post hoc* test. *p<0.05, **p<0.01, ***p<0.001, ****p<0.0001, ns, not significant.

The cytokine levels at baseline and day 7 are shown in **Figures 1**. For the majority of cytokines, significant changes in levels occurred between day 0 and day 7 and the pattern of change was very consistent among patients. The changes can be grouped as follows: Levels of IL-1ß, IL-2, IL-4, IL-5, IL-10, IL-12 (p70), IL-13, IL-17, IFN-γ, Eotaxin, GM-CSF and MIP-1α significantly increased from baseline to day 7, whereas IL-1RA, IL-7, IL-15, TNF-α, basic FGF and G-CSF showed a significant decrease on day 7 compared to baseline. Other cytokines numerically increased (IL-6, MIP-1ß) or decreased (IL-9, IP-10, VEGF, PDGF, RANTES, MCP-1), however, their medians on day 0 and 7 did not significantly differ after correction for multiple comparisons (**Figures 1**).

**Figure 2:**
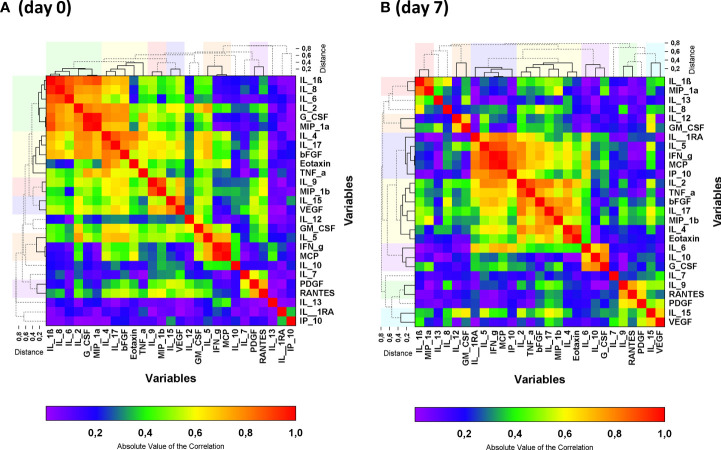
Heat map of the absolute values of the correlation matrix at baseline (day 0) **(A)** and on day 7 **(B)** of all patients. Clustered cytokines are indicated by the solid lines in the dendrograms and the clusters are indicated by the colors above and beside the graph. Non-clustered cytokines are shown by the dashed lines in the dendrogram. Clusters were formed by the unweighted group method. Cluster reports for the absolute values of the correlation matrix are presented in **Table 2A**.

**Figure 3:**
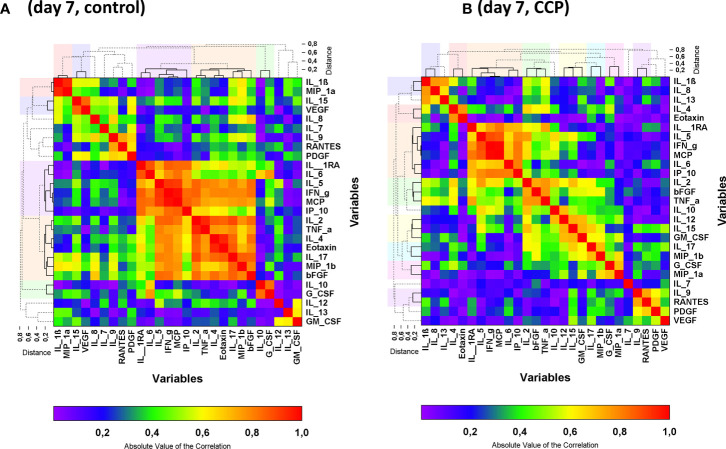
Heat map of the absolute values of the correlation matrix on day 7 stratified by randomization group: control **(A)** and CCP **(B)**. Clustered cytokines are indicated by the solid lines in the dendrograms and the clusters are indicated by the colors above and beside the graph. Non-clustered cytokines are shown by the dashed lines in the dendrogram. Clusters were formed by the unweighted group method. Cluster reports for the absolute values of the correlation matrix are presented in **Table 2B**.

**Figure 4:**
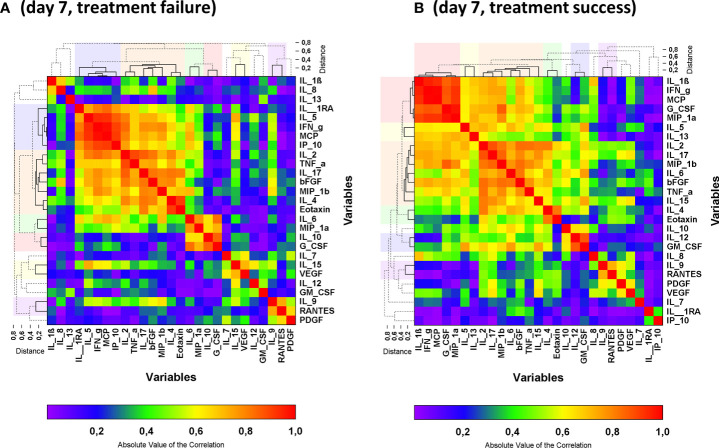
Heat map of the absolute values of the correlation matrix on day 7 in an analysis including both control group and CCP group stratified by reaching the primary endpoint on day 21: failure **(A)** and success **(B)**. Clustered cytokines are indicated by the solid lines in the dendrograms and the clusters are indicated by the colors above and beside the graph. Non-clustered cytokines are shown by the dashed lines in the dendrogram. Clusters were formed by the unweighted group method. Cluster reports for the absolute values of the correlation matrix are presented in **Table 2B**.

Cytokine changes in the disease course of COVID-19 can be influenced by many factors, e.g. the natural history of the disease, risk factors, severity of the disease, and therapeutic interventions. To analyze the effects of standard of care or CCP, we have analyzed the cytokine pattern at baseline and day 7 (**Figure 2**) and the latter analysis was also stratified by allocation of patients to the control group or convalescent plasma group (**Figure 3**) and by primary endpoint on day 21 (including both convalescent plasma group and control group, stratified by failure or success)(**Figure 4**). Some markers were strongly correlated as shown in the heat map of the adjusted correlation matrix (**Figure 2**). Rows and columns are sorted in the order suggested by a hierarchical clustering of the correlation matrix (**Table 2**). At day 0 in particular the following clusters were noted: (i) IL-1ß, IL-8, IL-6, IL-2, G-CSF and MIP-1α; (ii) IL-4, IL-17, bFGF, Eotaxin and TNF-alpha (iii) IL-9 and MIP-1ß, (iv) IL-15 and VEGF, (v) IL-5, IFN-γ and MCP-1; (vi) PDGF-BB and RANTES (**Figure 2**; **Table 2A**). Low correlation with all other cytokines was noted for IL-1RA, IL-7, IL-10, IL-12, IL-13, GM-CSF and IP-10 (**Figure 2A**; **Table 2A**). At day 7 the heat map changes substantially and the following clusters were formed: (i) IL-1ß and MIP-1α; (ii) IL-12 and GM-CSF; (iii) IL-1RA, IL-5, IFN-γ, MCP-1 and IP-10; (iv) IL-2, TNF-α, bFGF, IL-17, MIP-1ß, IL-4 and Eotaxin; (v) IL-6, IL-10, G-CSF; (vi) IL-9 and RANTES, (vii) IL-15 and VEGF. Low correlation with all other cytokines were noted for IL-7, IL-8, IL-13 and PDGF-BB (**Figure 2B**; **Table 2A**)

### Cytokine levels on day 7 and their association with randomization group and outcome

The pattern in the correlation matrix on day 7 differed between the control group and the CCP group (**Figures 3A, B**). IL-1RA, IL-5, IL-6, IFN-γ, IP-10, MCP-1 were grouped together in the hierarchical clustering approach both in the control group and the CCP group on day 7 (**Figure 3** and **Table 2B**). Also, the correlation between IL-2, bFGF and TNF-α as well as between IL-4 and Eotaxin was seen in both randomization groups. However, correlations between other cytokines changed substantially from baseline to day 7. Overall, the correlation heat maps indicate an impact of treatment (with/without CCP) and a correlation with outcome (treatment success or failure) which is less evident at the level of individual cytokines.

Also, when comparing patients who did or did not meet the primary endpoint at day 21, there was a clear difference in the pattern in the correlation matrix on day 7 (**Figure 4**; **Table 2B**). While IL-9 and RANTES, as well as IL-4, Eotaxin and IL-2, IL-17, bFGF, MIP-1ß and TNF-α were grouped together by the hierarchical clustering irrespective of primary outcome other clusters differ. Differences in the correlation matrix exist with a prominent cluster of IL-1ß, IFN-γ, MCP-1, G-CSF, MIP-1α in patients with treatment success and a prominent cluster of IL-1RA, IL-5, IFN-γ, IP-10 and MCP-1 in patients with treatment failure (**Figure 4**; **Table 2B**).

### Cytokine levels and clinical outcome: Receiver operating characteristics analysis

**Figure 5:**
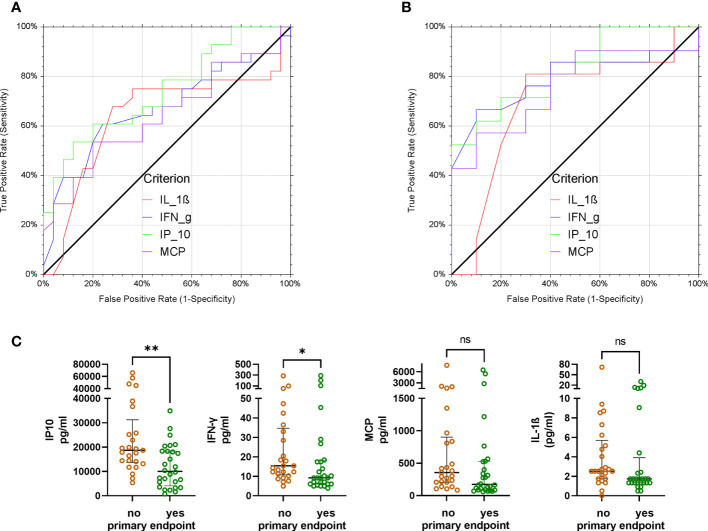
Receiver operating characteristics analysis of day 0 levels of IP-10, IFN-γ, IL-1ß and MCP-1 and primary endpoint (failure versus success on day 21). Low levels of these cytokines on day 0 indicate a positive condition, i.e. patients reached the primary endpoint.**(A)** All patients (irrespective of allocation to randomization group). Area under the curve (AUC) and p-values for AUC >0.5 were as follows: IP-10: AUC 0.74; p=0.0002; IFN-γ: AUC 0.67, p=0.013; MCP-1: AUC 0.64, p=0.03; IL-1ß: AUC 0.64, p=0.04).**(B)** Patients in the CCP group Area under the curve (AUC) and p-values for AUC >0.5 were as follows: IP-10: AUC 0.82; p<0.0001; IFN-γ: AUC 0.79, p=0.0002; MCP-1: AUC 0.76, p=0.002; IL-1ß: AUC 0.70, p=0.04). **(C)** Comparison of day 0 levels of IP-10, IFN-γ, MCP-1 and IL-1ß (from left to right) between patients not reaching the primary endpoint (brown symbols) or reaching the primary endpoint on day 21 (green symbols). Groups were compared by Mann-Whitney test. *p<0.05, **p<0.01, ***p<0.001, ****p<0.0001.

Then we analyzed whether the cytokines on day 0 and day 7 can be used as marker to predict the primary outcome, i.e. survival on day 21 and no longer fulfilling criteria of severe COVID-19. ROC analysis for the primary outcome was performed for all cytokines. Low levels of IP-10, IFN-γ, MCP-1 and IL-1ß on day 0 were predictive of reaching the primary endpoint on day 21 with an AUC of 0.74, 0.67, 0.64 and 0.64 (Fig 5A). A subgroup analysis by treatment group (control group vs CCP group) revealed that in the control group day 0 levels of none of these four cytokines were predictive of reaching the primary endpoint (not shown), whereas in the CCP group the levels of all four cytokines were predictive of reaching the primary endpoint (Fig 5B). The bivariate comparison of day 0 levels of IP-10, IFN-γ, MCP-1 and IL-1ß, between patients who met or did not meet the primary endpoint on day 21 is shown in **Figure 5C**.

For none of the measured cytokines increased levels at day 0 were associated with a better primary outcome in the ROC analyses.

**Figure 6:**
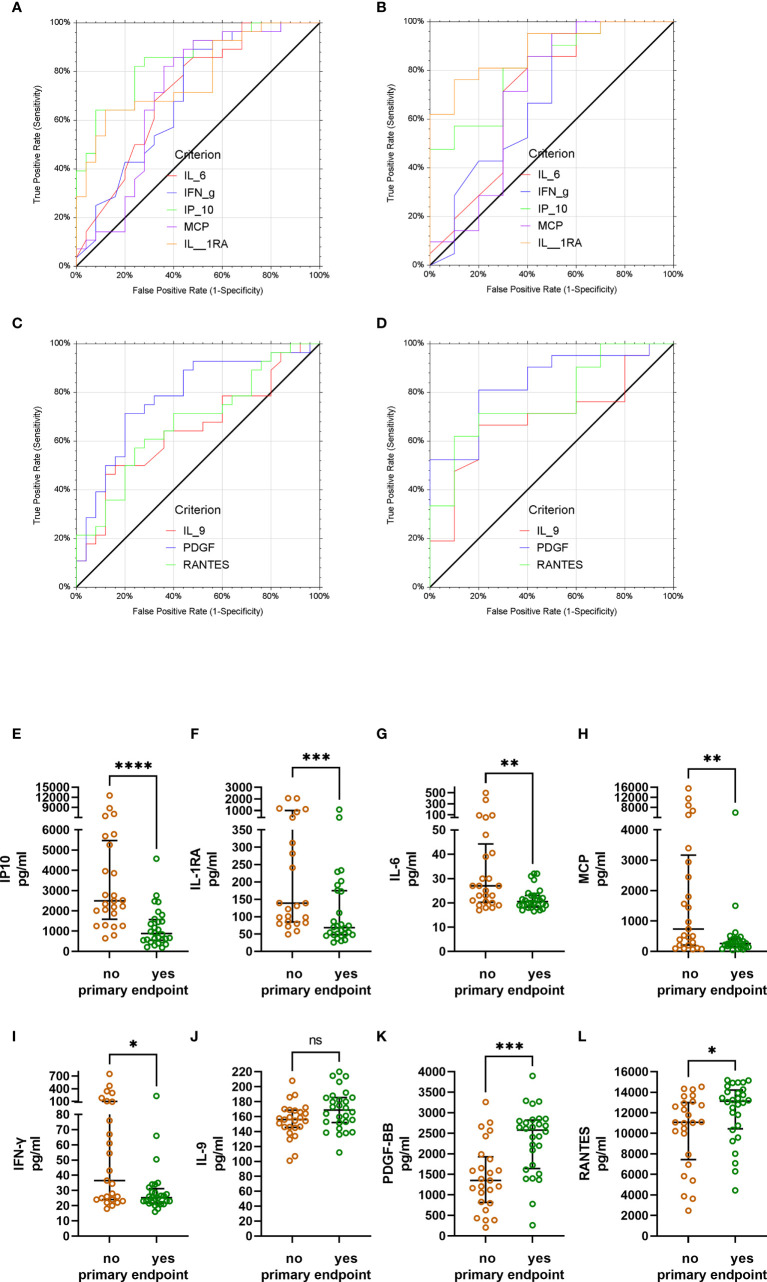
Receiver operating characteristics analysis of day 7 cytokine levels and primary endpoint (failure vs. success on day 21). **(A)** All patients (irrespective of allocation to randomization group). Low levels of IP-10, IL-1RA, MCP-1, IL-6 and IFN-γ on day 7 indicate treatment success. Area under the curve (AUC) and p-values for AUC >0.5 were as follows: IP-10: AUC 0.85; p<0.0001; IL-1RA: AUC 0.78, p<0.0001; IL-6: AUC 0.72, p=0.0012; MCP-1: AUC 0.71, p=0.0033; IFN-γ: AUC 0.70, p=0.0038). **(B)** Patients in the CCP group. Low levels of IP-10, IL-1RA, MCP-1 and IL-6 on day 7 indicate treatment success. AUC and p-values for AUC >0.5 were as follows: IP-10: AUC 0.82; p<0.0001; IL-1RA: AUC 0.89, p<0.0001; IL-6: AUC 0.71, p=0.0345; MCP-1: AUC 0.70, p=0.0499; IFN-γ: AUC 0.69, p=0.0516). **(C)** All patients (irrespective of allocation to randomization group). High levels of PDGF-BB, RANTES and IL-9 on day 7 indicate treatment success. AUC and p-values for AUC >0.5 were as follows: PDGF-BB: AUC 0.78; p<0.0001; RANTES: AUC 0.68, p=0.0080; IL-9: AUC 0.65, p=0.0260). **(D)** Patients in the CCP group. High levels of PDGF-BB, RANTES and IL-9 on day 7 indicate treatment success. AUC and p-values for AUC >0.5 were as follows: PDGF-BB: AUC 0.84; p<0.0001; RANTES: AUC 0.77, p=0.0012; IL-9: AUC 0.69, p=0.0279). **(E-L)** Bivariate comparison of day 7 levels of IP-10, IL-1RA, IL-6, MCP-1, IFN-γ, IL-9, PDGF-BB and RANTES between the patients not reaching the primary endpoint (brown symbols) or reaching the primary endpoint on day 21 (green symbols). Groups were compared by Mann-Whitney test. *p<0.05, **p<0.01, ***p<0.001, ****p<0.0001. ns, not significant.

Low levels of IP-10, IL-1RA, IL-6, MCP-1 and IFN-γ (**Figure 6A**) and high levels of IL-9, PDGF-BB and RANTES (**Figure 6C**) on day 7 were predictive of reaching the primary outcome. A subgroup analysis by randomization group (control group versus CCP group) again revealed a substantial difference between these groups. In the control group only, low levels of IP-10 on day 7 were predictive of reaching the primary outcome (not shown). In the CCP group low levels of IP-10, IL-1RA, IL-6 and MCP-1 on day 7 (**Figure 6B**) as well as high levels of IL-9, PDGF-BB and RANTES on day 7 (**Figure 6D**) were predictive of meeting the primary endpoint on day 21. The bivariate comparison of day 7 levels of IP-10, IL-1RA, IL-6, MCP-1, IFN-γ, IL-9, PDGF-BB and RANTES between the patients who met or did not meet the primary endpoint on day 21 is shown in **Figures 6E-L**.

A key secondary endpoint in the clinical trial was the time to clinical improvement which was defined as an improvement by at least 2 points on the 8-point WHO ordinary severity scale (10, 11). We analyzed whether the cytokines levels on day 0 and day 7 can be used to predict a rapid clinical improvement (i.e. time to clinical improvement ≤ median).

ROC analysis demonstrated that low levels of IP-10 (AUC 0.73, p=0.0046), MCP-1 (AUC 0.71, p=0.0088), IL-6 (AUC 0.71, p=0.0059), IL-10 (AUC 0.67, p=0.0236) and IL-1ß (AUC 0.67, p=0.0308) at baseline were predictive of a shorter time to clinical improvement. Again, for none of the cytokines were increased levels on day 0 associated with a shorter time to clinical improvement in the ROC analysis.

Low levels of IP-10 (AUC 0.76, p=0.0001, IL-1RA (AUC 0.73, p=0.0023), MCP-1 (AUC 0.71, p=0.0021) and IFN-γ (AUC 0.64, p=0.0409) on day 7 and high levels of PDGF-BB (AUC 0.70; p=0.0066) and RANTES (AUC 0.67, p=0.0229) on day 7 were predictive of early clinical improvement. In a subgroup analysis by treatment group, only IP-10 and IL-1RA were predictive of early clinical improvement in all subgroups.

### Cytokine levels and clinical outcome: Multivariate analyses

**Figure 7:**
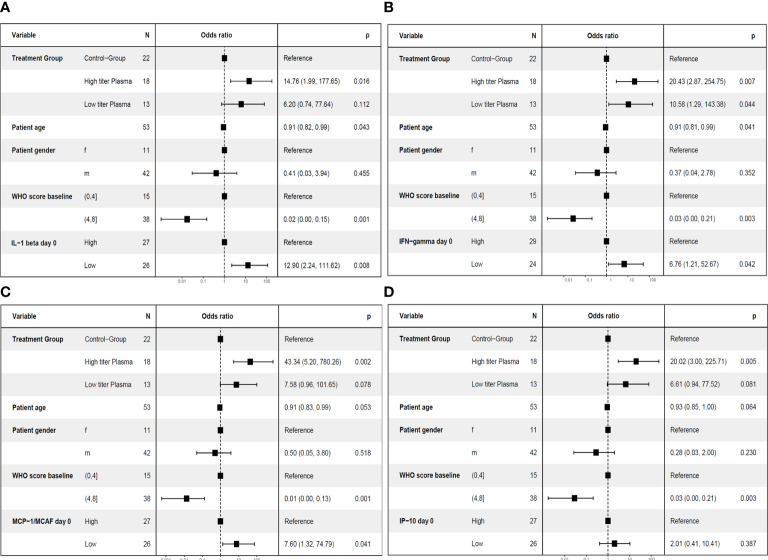
Multivariate analyses of primary endpoint (survival and no longer meeting criteria for severe COVID-19 on day 21) including cytokine levels on day 0. The following variable were included in the model: Treatment group (control group, high titer plasma, low titer plasma), age (as continuous variable), gender (female (f)) and male (m), baseline WHO Severity Score (≤4 vs. > 4) (11) and the level of the respective cytokine on day 0 (≤ median (“low”) versus > median (“high”)). One cytokine each was included in the models: IL-1ß **(A)**, IFN-γ **(B)**, MCP-1 **(C)** or IP-10 **(D)**.

**Figure 8:**
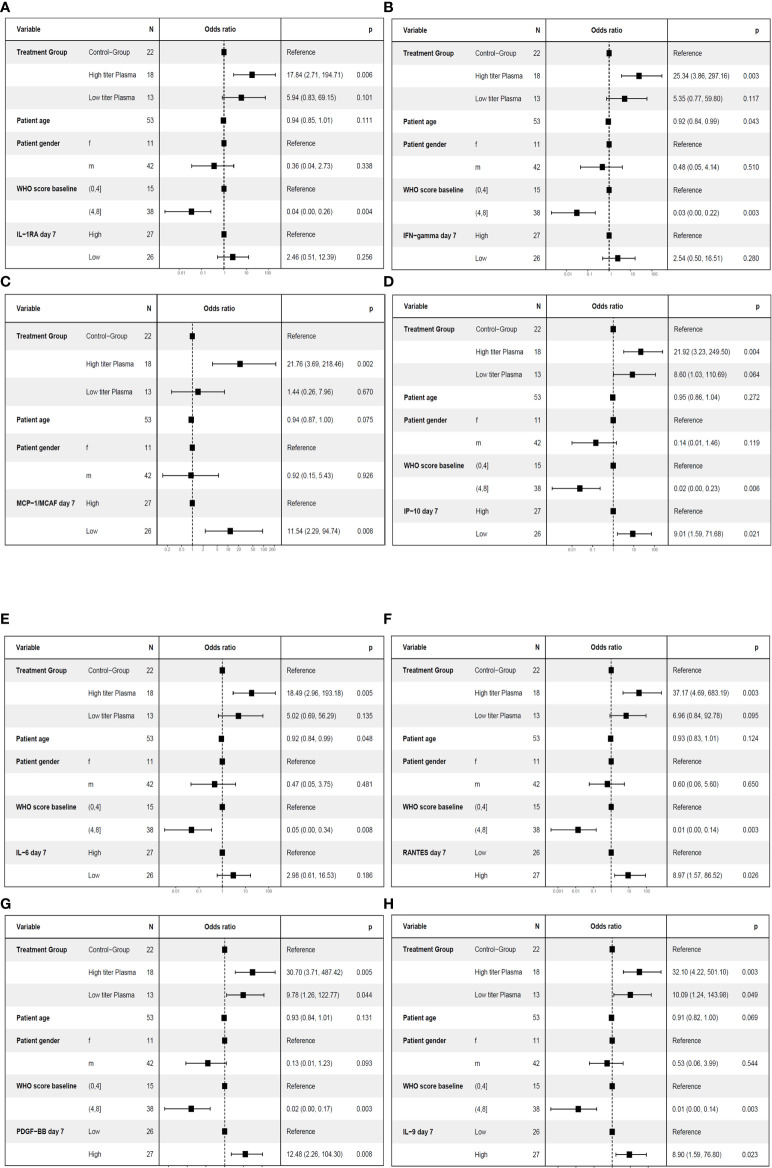
Multivariate analyses of primary endpoint (survival and no longer meeting criteria for severe COVID-19 on day 21) including cytokine levels on day 7. The following variable were included in the model: Treatment group (control group, high titer plasma, low titer plasma), age (as continuous variable), gender, baseline WHO Severity Score (≤4 vs. > 4) (11) and the level of the respective cytokine on day 7 (≤ median (“low”) versus > median (“high”)). One cytokine each was included in the models: IL-1RA **(A)**, IFN-γ **(B)**, MCP-1 **(C)**, IP-10 **(D)**, IL-6 **(E)**, RANTES **(F)**, PDGF-BB **(G)** or IL-9 **(H)**.

**Figure 9:**
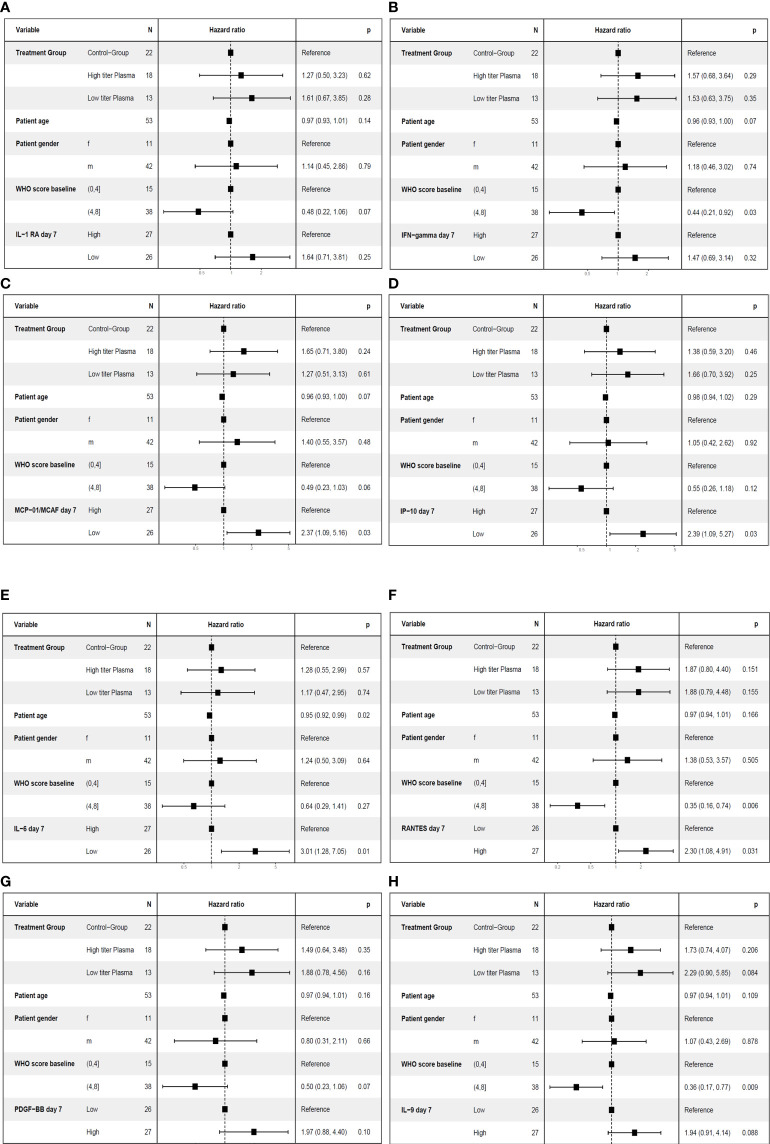
Multivariate analyses of the key secondary endpoint time to clinical improvement (≤ median vs. > median) including cytokine levels on day 7. The following variables were included in the model: Treatment group (control group, high titer plasma, low titer plasma), age (as continuous variable), gender, baseline WHO Severity Score (≤4 vs. > 4) (11) and the level of the respective cytokine on day 7 (≤ median (“low”) versus > median (“high”)). One cytokine each was included in the models: IL-1RA **(A)**, IFN-γ **(B)**, MCP-1 **(C)**, IP-10 **(D)**, IL-6 **(E)**, RANTES **(F)**, PDGF-BB (panel **G**) or IL-9 (panel **H**).

**Figure 10:**
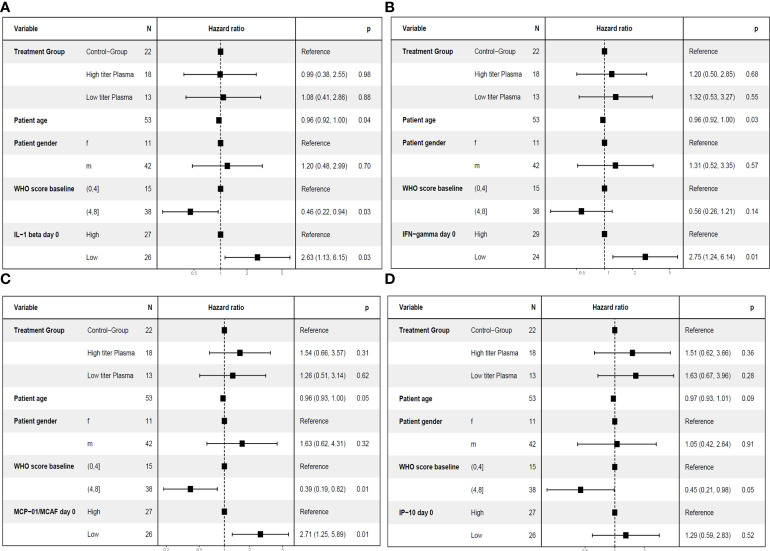
Multivariate analyses of the key secondary endpoint time to clinical improvement (≤ median vs. > median) including cytokine levels on day 0. The following variable were included in the model: Treatment group (control group, high titer plasma, low titer plasma), age (as continuous variable), gender, baseline WHO Severity Score (≤4 vs. > 4) (11) and the level of the respective cytokine on day 0 (≤ median (“low”) versus > median (“high”)). One cytokine each was included in the models: IL-1ß **(A)**, IFN-γ **(B)**, MCP-1 **(C)** or IP-10 **(D)**.

In this population of patients with severe COVID-19 outcome can be affected by various factors, in particular by the severity of disease at baseline, the treatment of COVID-19 and demographic characteristics (age, gender). As shown above, the cytokine pattern on day 7, i.e. after transfusion of three units of CCP, differed substantially between control group and CCP group (**Figure 3**). Furthermore, the prediction of the primary endpoint in the ROC analysis differed between control and CCP group. Both aspects emphasize the impact of the randomization group on outcome. Therefore, we also examined the association between cytokine levels at baseline or day 7 and the primary endpoint in a multivariate analysis, which included the study treatment, the WHO COVID-19 Severity Score at baseline (Score 0 to 4 vs. Score 5 to 8), age and gender. A pre-specified subgroup analysis of the CAPSID Trial has shown a difference among patients who have received either a low cumulative amount of neutralizing SARS-CoV-2 antibodies (“low titer plasma”) or a high cumulative amount of neutralizing SARS-CoV-2 antibodies (“high titer plasma”) (10). Therefore, treatment in this multivariate analysis has been stratified in control group, high titer and low titer plasma (**Figures 7**– **10**). The multivariate analyses demonstrated a significant higher odds ratio for a favorable outcome, i.e. reaching the primary endpoint, in the CCP treated patients. In contrast, patients with severe disease as indicated by WHO COVID-19 Severity Score >4 had poor outcome. In these multivariate models, the level of IL-1ß, IFN-γ and MCP-1 at baseline (comparing equal to or below median vs. above median) were significantly associated with both treatment success (**Figures 7A-C**) and short time to clinical improvement (**Figures 9A-C**). Low IP-10 levels on day 0 which were significant in the ROC analysis, were not significant in the multivariate model for treatment success (**Figure 7D**).

Lower levels of MCP-1 and IP-10 on day 7 and higher levels of PDGF-BB, RANTES and IL-9 on day 7 were associated with a significant higher odds ratio for treatment success (**Figures 8C, D, F-H**). Lower levels of IL-1RA, IFN-γ, and IL-6 on day 7 which were significant in the ROC analysis were not significant in the multivariate model for treatment success (**Figures 8A, B, E**). Low levels of IP-10, MCP-1 and IL-6 on day 7 and high levels of RANTES on day 7 were also significant in the multivariate model for short time to clinical improvement (**Figures 10C-F**). In contrast, low levels of IL-1RA and IFN-γ (**Figures 10A, B**) or high levels of PDGF-BB or IL-9 (**Figures 10G, H**) were not significantly associated with shorter time to clinical improvement. Low levels of IP-10 on day 7 and high levels of PDGF on day 7 were significant in the multivariate model for overall survival (not shown).

A summary of the results in the various models for the primary endpoint and the key secondary endpoint is provided in **Table 3**.

**Table 3:** Summary of cytokines with significant results for cytokine levels at baseline and on day 7 for the primary endpoint or the key secondary endpoint “time to clinical improvement” in both the ROC analysis and the multivariate models.

	Primary Endpoint:(survival and no longer severe COVID-19 on day 21)		Key secondary endpoint:time to clinical improvement	
	Level Baseline	Level day 7	Level Baseline	Level d7
**Low levels of**				
** IL-1ß**	ROC MVA		ROC MVA	
** IP-10**	ROC	ROC MVA	ROC	ROC MVA
** IFN-γ**	ROC MVA	ROC	MVA	ROC
** MCP-1**	ROC MVA	ROC MVA	ROC MVA	ROC MVA
				
**High levels of**				
** IL-9**		ROC MVA		
** PDGF-BB**		ROC MVA		ROC
** RANTES**		ROC MVA		ROC MVA

ROC indicates that the respective parameter is significant in the ROC analysis (see **Figure 5**, **6** and text)

MVA indicates that the respective parameter is significant in the multivariate analysis (**Figures 7**, **8** for primary endpoint, **Figures 10**, **9** for key secondary endpoint).

Primary endpoint: ROC analysis in **Figures 5**, **6**; multivariate analysis **Figure 7**, **8**; key secondary endpoint: multivariate analysis **Figures 10**, **9**.

## Discussion

Pathophysiology of COVID-19 has been linked to an uncontrolled systemic inflammatory response. It has been demonstrated that levels of many cytokines are increased in the acute phase of SARS-CoV-2 infection. A correlation of this hyperinflammation and cytokine release syndrome with the severity and outcome of COVID-19 has been reported (6, 20–22). Increased levels of several cytokines have been associated with severity (IL-6, IL-1RA, IL-10, IL-15, IL-27, G-CSF, M-CSF, IP-10, TNF-α, MIG, MCP-1) (5–7, 15–19). A clustering of four immune signatures representing growth factors (e.g. PDGF, VEGF), type 2/3 cytokines, mixed type 1/2/3 cytokines and chemokines have been reported in conjunction with disease trajectories (21). Also in our patient cohort from the clinical trial CAPSID (10) we observed a clustering of cytokines, which was, however, different from the clustering as proposed by Lucas et al. (21). The differences are most likely due to different severity and different treatment of the patient cohorts and differences in the overall profile of investigated cytokines.

For the majority of cytokines, we observed a pattern of either clear increase or decrease between day 0 and day 7 in the majority of patients. Dynamic changes of various cytokines has been reported by others –interpreted as “early” cytokines (decrease from baseline) and “late” cytokines (increase) (6). Kelymenov et al. reported a pattern with initial high levels of TNF, IL-6, IL-18, IL-27, IL-15, IFN-α2, GM-CSF, G-CSF, M-CSF, IP-10, MIG, MCP-1, GRO-α (interpreted as early cytokines representing markers of innate immune response and type 1 immunity). In contrast, the “late” cytokines, characteristic of type 2 immunity (IL-4, IL-5, IL-13), were increased in this study seven days after onset of symptoms (6). While we observed a significant change between baseline and day 7 which was consistent across the study population, we could not confirm a uniform pattern of early cytokines representing innate and type 1 immunity versus late cytokines representing type 2 immunity. Some changes suggest a switch towards a type 2 signature on day 7 (increase of IL-4, IL-5, IL-10, IL-13). However, also type 1 cytokines increase from baseline to day 7 (increase of IFN-γ, IL-2, IL-12). IL-1RA, IL-7, IL-15, TNF-α, bFGF and G-CSF decreased from baseline to day 7. This might be due to the study population of the CAPSID Trial. Based on the eligibility criteria of this clinical trial, our study included only patients with severe COVID-19. Notably, the intervention with CCP in the experimental group seems to have changed the time course and pattern of cytokine levels. While some of the cytokines were grouped together in the hierarchical correlation matrix on day 7 both in the control group and the CCP group (e.g. IL-5, IFN-γ, MCP-1 and IP-10; or IL-2 and TNFα), correlation between others differed between control and CCP group (e.g. IL-1ß and MIP-1α, or IL-1RA and IL-6)(**Figure 3**; **Table 2B**).

Due to the strong association of cytokine levels with the severity of the disease it is reasonable to investigate the prognostic significance of the elevated cytokines (23, 24). Ozger *et al.* reported that higher levels of IL-6, IL-7, IL-10, IL-15, IL-27, IP-10, MCP-1 and G-CSF were predictive for mortality (25), in particular IL-6, IL-10, IL-7 and G-CSF had higher sensitivity and specificity in predicting mortality and hospital admission (25). Kelymenov *et al.* found that the levels of nine cytokines, TNF-α, IL-10, IL-6, IP-10, M-CSF, G-CSF, GM-CSF, IFN-α2 and MIG (Monokine induced by Gamma-Interferon; CXCL9) at 4 to 6 days after symptom onset are good predictors of requirement intensive care treatment (6). Ashrafzadeh-Kian *et al.* identified elevated IL-6 as best predictor of the need for hospitalization and length of hospital stay (1). In this regard, others have suggested TNF-α, IL-6, IL-10 and IL-1RA as markers predicting outcome of COVID-19 (20, 22, 26, 27). Herr et al. reported increased IL-1A, IL-8, IL-10, MCP-1 and SCF levels in patients that died (28).

Thus, our analysis in part confirms these findings of other groups (**Table 3**). Particular emphasis was placed on cytokines, which were significant in both the ROC analysis and the multivariate analysis, as well as across various clinical endpoints (primary endpoint and key secondary endpoint of the clinical trial CAPSID)(summary in **Table 3**): low levels of IP-10 on day 0 and low levels of IP-10, IL-1RA, IL-6 on day 7 were predictive of treatment success in the ROC analysis success. However, in contrast to other reports we highlight the predictive role of MCP-1, IP-10 and RANTES (**Table 3**). We identified low levels of IL-1ß and MCP-1 at baseline as strong predictors of a favorable outcome. This has been confirmed by ROC analyses and multivariate models for various endpoints (treatment success according to the primary outcome definition of the CAPSID trial, the time to clinical improvement as key secondary outcome). Here low levels of MCP-1 and IP-10 on day 7 as well as high levels of RANTES on day 7 were associated with better outcome as demonstrated by both ROC analyses and multivariate analyses for treatment success and shorter time to clinical improvement. Thus, our study provides a strong signal for the predictive significance of IL-1ß, MCP-1, IP-10 and RANTES on day 0 and 7, resp. and we suggest to include IL-1ß and MCP-1 on day 0, and MCP-1, IP-10 and RANTES on day 7, in the evaluation of cytokine profiles in future studies for further validation in larger cohorts and various treatment settings.

The source of these cytokines cannot be determined from our study. The pattern is however consistent with activation of the inflammasome and monocyte activation which has been shown by several studies: SARS-CoV-2 Viroporin-3a was sufficient to induce the NLRP3 inflammasome activation and IL-1ß production in macrophages stimulated by LPS (29). It has been shown that SARS-CoV-2 engages inflammasome and triggers pyroptosis in human monocytes, experimentally infected, and from patients under intensive care (30, 31). Pyroptosis associated with caspase-1 activation, IL-1ß production, gasdermin D cleavage, and enhanced pro-inflammatory cytokine levels in human primary monocytes (30). The infection of monocytes in patients with COVID-19 depends on the uptake of antibody-opsonized virus by Fcγ receptors (31). This might –at least in part- explain the difference cytokine patterns which we observed between control group and CCP group in our trial. In addition, the increased IL-1ß production might be an indicator of cell death in the airway epithelium due to viral cytopathic effects (32, 33). Since SARS-CoV-2 S protein can up-regulate angiotensin converting enzyme (ACE2) and MCP-1 in endothelial cells (34) increased levels of MCP-1 might also indicate endothelial dysfunction.

Type 1 interferons have not been included in our analysis. Despite its fundamental role in health and disease (e.g. in viral infections or autoimmunity such as systemic lupus erythematosus), the direct quantification of type I IFNs is still challenging. Rather than a direct measurement of type I interferons its influence on immune cells is accessed by interferon-induced transcripts (IFITs) in whole blood or PBMCs (35). Other surrogate parameters of type I interferon signature such as CD169 or STAT1 have been used (36, 37). Others have shown that impaired INF-type I response might lead to increased virus replication and dysregulated pulmonary inflammation. In particular, there is evidence that either inborn errors of type I IFN immunity (38) or autoantibodies against IFN-type I (39) can account for life-threatening COVID-19 in a subgroup of patients. Further humans express 12 IFN-alpha subtypes, which have different properties (40). Analysis of autoantibodies, IFN-alpha-subtypes and IFITs was not within the scope of our study.

Many biomarker studies including ours investigated soluble circulating cytokines, chemokines and growth factors. Other studies used gene expression profiles in peripheral blood cells or tissues, e.g. nasopharyngeal samples or lung biopsies, or circulating microRNA sigantures to predict COVID-19 prognosis (41–45). Flower-Lose is a unique marker for suboptimal or unfit status of cells in many tissues or organ systems. hFwe-Lose can precede the host immune response to infection and accurately predicts outcomes in COVID-19 patients (45). Another approach used targeted transcriptomics of lung biopsies to provide a transcriptional landscape of COVID-19 in the lung (42). A significant upregulation of genes associated with inflammation, type I interferon production, coagulation and angiogenesis was observed in the lungs of COVID-19 patients compared to non-infected controls (42). Circulating microRNA signatures with high concentration of miRNA targeting antiviral response and low miRNAs targeting pro-inflammatory factors discriminated severe from mild/moderate COVID-19 (41).

The observed combination of predictive factors at baseline is compatible with the hypothesis that lower levels of inflammasome activation and monocyte activation and less endothelial dysfunction are associated with better clinical outcome. The role of IL-1ß as predictive markers raises the question whether its inhibition could be beneficial. Some trials have suggested clinical improvement in COVID-19 patients treated with Interleukin-1 blocking agents, however a recent COCHRANE Review concluded that there is no evidence for an important beneficial effect of IL-1 blocking agents. The evidence, however, is uncertain or very uncertain for several outcomes and there are many registered trials of anakinra and canakinumab with no results publicly available yet (46).

Previous studies have not yet shown that these factors (IL-1ß and MCP-1 on day 0 and MCP-1, IP-10 and RANTES on day 7) in particular have predictive significance in combination. One study on longitudinal cytokine profiling concluded that a combination of CCL5 (RANTES), IL-1RA and IL-10 at week 1 may predict outcome (22). High levels of IL-1ß and IL-18 as indicators of inflammasome activation have been associated with the risk of admission to intensive care unit and death (21). A significant increase of MCP-1 has been noted in severe/critically ill patients but no predictive significance for outcome was established in this study (16) while in other studies an association of high MCP-1 levels and admission to intensive care unit was observed (47). Another study found higher levels of IL-6, TNF-α, CCL2 (MCP-1), CCL5 (RANTES) in non-survivors at 0-72 hours before death (48). MCP-1 and soluble IL-2Rα were moderate predictors of the need for hospitalization in a cohort of patients with various disease severities in a study which identified elevated IL-6 as best predictor of severity of COVID-19 (1).

Thus, the cytokine patterns associated with clinical outcome in published studies partially overlap, but show also differences to our findings. This could be explained by several reasons: (i) In these studies, the severity of COVID-19 and the interval since infection or onset of symptoms varied greatly. Since there is a dynamic change of the cytokine profile during the clinical course of COVID-19, as also shown in our work, the timing of the cytokine measurements has great impact on results. (ii) It is known that gender, age, disease severity, comorbidities, and therapy are also important for outcome of COVID-19. The patient cohorts analyzed in prior cytokine studies differed in these additional prognostic factors, in some cases significantly. Also, in the previous studies, the endpoints investigated were different (requirement for intensive care treatment, mortality, length of hospital stay and others) and the statistical methods differed, mostly only ROC analyses were used. Our proposal to consider especially IL-1ß, MCP-1, IP-10, and RANTES as predictive markers for further studies is based not only on ROC analyses but also on multivariate models considering other prognostic factors (treatment, age, gender, clinical severity). Furthermore, the predictive significance of these factors has been confirmed across several meaningful clinical endpoints (treatment success which is a composite endpoint of survival and no longer fulfilling criteria of severe COVID-19 on day 21; short time to clinical improvement). (iii) Furthermore, the impact of treatment needs to be considered. The cytokines can be grouped into clusters by unsupervised cluster analysis. These clusters change over time and importantly are also influenced by the CCP treatment. The impact of CCP treatment is also evident in the ROC analyses in our study: while low levels of IL-1ß, IP-10, IFN-γ, and MCP-1 on day 0 are predictive of treatment success and short time to clinical improvement in the total study cohort and in the CCP group, this relationship is not significant in a subgroup analysis of the control group. The same applies to low levels of IL-6, IFN-γ, MCP-1 and IL-1RA on day 7 which were no longer significant in a subgroup analysis of control group patients.

The investigational drug in the CAPSID trial was CCP. However, the same impact on cytokine course might apply to other anti-inflammatory or antiviral interventions. Therefore, all results on association between cytokine patterns and outcome need to be interpreted in the specific treatment context and might not be generalized. The significant impact of CCP on key regulators of inflammation is an important finding and supports the effect of CCP in severe COVID-19.

The strength of our study is the controlled setting of a clinical trial with pre-specified treatment and pre-specified definitions of outcomes and evaluation time points. This reduces the noise due to other influencing factors and allows predictive factors to be more precisely identified. On the other hand, this is also a limitation of our study, since the observed associations might only apply to the setting of CCP treatment. Our study included patients with severe COVID-19 which were predominantly male (10)– as in other studies (49–53).

Overall, it is important to establish predictive factors. Therefore, future studies should validate the predictive value of IL-1ß and MCP-1 at baseline and MCP-1, IP-10, and RANTES on day 7 in addition to other cytokines, in larger patient cohorts receiving standardized treatment, ideally in the context of controlled clinical trials. E.g., the ongoing COVIC-19 trial (NCT05271929; EudraCT 2021-006621-22) will further investigate the use of cytokine profiling as predictive biomarker.

## Data availability statement

The datasets presented in this article are not readily available because for research purposes, the original data are available upon request and after internal review. Requests to access the datasets should be directed to https://h.schrezenmeier@blutspende.de.

## Ethics statement

The studies involving human participants were reviewed and approved by Ethical Committee of the University of Ulm. The patients/participants provided their written informed consent to participate in this study.

## Author contributions

Author Tasks Design of the CAPSID-Trial: HS, SK, ES. Clinical Trial Coordination: SK. Patient Care: BG, DZ, MW, TW, KZ, JK, MB, MD, GP, PL, LE, HW, SZ, BJu. Clinical Trial Management: TA, BJa, MR, RL, HS, SK. Cytokine Measurements: ES, H-RA, TD. Data Analysis: DF, HS, ES. Writing the manuscript: HS, DF, ES, SK. All authors contributed to the article and approved the submitted version.

## Funding

Bundesministerium für Gesundheit (German Federal Ministry of Health): ZMVI1-2520COR802 and ZMI1-2521COR802. The entire study was funded by the German Federal Ministry of Health. The Ministry had no role in analyzing the data, writing the manuscript or deciding to submit it for publication. The clinical trial CAPSID is supported by the Bundesministerium für Gesundheit (“German Federal Ministryof of Health”)(ZMVI1-2520COR802) and. ZMI1-2521COR802.

## Acknowledgments

We thank all patients who participated in this trial. We thank the clinical research teams, physicians, study nurses and data managers in all clinical trial centers and the team of the CRO Alcedis.

## Conflict of interest

The authors declare that the research was conducted in the absence of any commercial or financial relationships that could be construed as a potential conflict of interest.

## Publisher’s note

All claims expressed in this article are solely those of the authors and do not necessarily represent those of their affiliated organizations, or those of the publisher, the editors and the reviewers. Any product that may be evaluated in this article, or claim that may be made by its manufacturer, is not guaranteed or endorsed by the publisher.
